# Darwin’s finches treat their feathers with a natural repellent

**DOI:** 10.1038/srep34559

**Published:** 2016-10-10

**Authors:** Arno Cimadom, Charlotte Causton, Dong H. Cha, David Damiens, Birgit Fessl, Rebecca Hood-Nowotny, Piedad Lincango, Alejandro E. Mieles, Erwin Nemeth, Elizabeth M. Semler, Stephen A. Teale, Sabine Tebbich

**Affiliations:** 1 Department of Behavioural Biology, University of Vienna, Vienna, Austria; 2 Charles Darwin Foundation, Puerto Ayora, Santa Cruz Island, Galápagos, Ecuador; 3 Department of Environmental and Forest Biology, College of Environmental Science and Forestry, State University of New York, Syracuse, USA; 4 Insect Pest Control Laboratory, Joint FAO/IAEA Division of Nuclear Techniques in Food and Agriculture, International Atomic Energy Agency, Vienna, Austria; 5 Health and Environment Department, AIT Austrian Institute of Technology GmbH, Tulln, Austria; 6 BirdLife Austria, Vienna, Austria

## Abstract

Darwin’s finches are highly innovative. Recently we recorded for the first time a behavioural innovation in Darwin’s finches outside the foraging context: individuals of four species rubbed leaves of the endemic tree *Psidium galapageium* on their feathers. We hypothesised that this behaviour serves to repel ectoparasites and tested the repellency of *P. galapageium* leaf extracts against parasites that negatively affect the fitness of Darwin’s finches, namely mosquitoes and the invasive hematophagous fly *Philornis downsi*. Mosquitoes transmit pathogens which have recently been introduced by humans and the larvae of the fly suck blood from nestlings and incubating females. Our experimental evidence demonstrates that *P. galapageium* leaf extracts repel both mosquitoes and adult *P. downsi* and also inhibit the growth of *P. downsi* larvae. It is therefore possible that finches use this plant to repel ectopoarasites.

Darwin’s finches are highly adaptable and able to cope with the unpredictable characteristics of the Galápagos Archipelago. They show an extraordinary number and diversity of feeding innovations, including consumption of novel food types and foraging behaviours (reviewed in ref. 1). These feeding innovations are novel in the sense that such behaviour patterns are unusual for passerines and it is likely that they evolved after their ancestors colonised the islands. It has been proposed that the ability to innovate so readily has contributed to the adaptive radiation of this clade^1^. Since the permanent colonisation of the Islands by humans, Darwin’s finches have been exposed to a new set of risks and challenges, in particular exotic parasites and pathogens that reduce the survival and reproductive success of the birds^2^,^3^,^4^,^5^. Here we report for the first time an innovation of this species group that is outside the feeding context: in 2012 we observed a warbler finch (*Certhidea olivacea*) tearing off the tips of leaves of the endemic Guayabillo tree, *Psidium galapageium,* and rubbing them on its feathers. Over the last four years, we have observed ten incidents of this behaviour in four species of Darwin’s finches (see Table 1). We have identified two different methods: (1) sponge method, the bird threads a piece of leaf through its feathers and (2) lotion method, the bird chews the leaf first and applies the mashed leaf to its feathers. In all instances, the birds used *P. galapageium* leaves, although this tree species occurs at low densities in the highlands of Santa Cruz Island where the observations were made.

Topical application is taxonomically widespread in birds but observations are rare and anecdotal^6^,^7^,^8^,^9^. Birds have mostly been observed applying arthropods, predominately ants, to their feathers and thus this behaviour is called “active anting”. Reports of birds applying plants to their feathers are rare; only a few instances have been recorded^10^,^11^,^12^. Suggested functions of such behaviour include control of ectoparasites, fungal or bacterial infections^12^,^13^,^14^,^15^ (but see ref. 16), and/or soothing the skin during moulting^7^. All observations of the application of *P. galapageium* leaves were made during the breeding season, when Darwin’s finches do not moult^17^ thus, soothing is unlikely to be the primary function of this behaviour during this period. We hypothesised that Darwin’s finches use the leaves of *P. galapageium* to repel ectoparasites and tested the repellent effect of these leaves against parasitic insects that reduce the fitness of Darwin’s finches, namely mosquitoes and the recently introduced parasitic fly *Philornis downsi*. Several mosquito species are native to the Galápagos but others have been introduced (e.g., *Culex quinquefasciatus*, *Aedes aegypti*^18^). More importantly, the appearance in the Galápagos of novel mosquito-borne pathogens such as avian pox is increasingly affecting the survival of Darwin’s finches^19^.

The reproductive success of Darwin’s finches is strongly reduced by the larval stage of *P. downsi*, a recently introduced, obligate bird parasite (reviewed in refs 20,21). The first larval stage is mainly found in the chicks’ nostrils. The second and third larval stages live in the bottom of the nest, pierce the skin of the chicks, and consume their blood^22^. However, for the first time in 2012 and again in 2014 and 2015 we recorded *P. downsi* larvae in nests that had been abandoned during incubation and in which no chicks had ever been present. As Darwin’s finches never re-use nests (personal observation) the larvae could not have hatched during a previous breeding attempt. Previously larvae had been found only in nests with hatchlings and never in nests containing unhatched eggs^3^,^21^,^23^. A total of 21 nests which had been abandoned during incubation were examined between 1998 and 2010. As this sample size is small it is possible that previous studies overlooked larvae in incubating nests. However, infestation during the incubation phase could also represent a gradual change of the parasite’s life cycle. In 2012 pupae and larvae of all developmental stages were found in 88% of abandoned warbler finch nests (15 out of 17 nests) and in 22% of abandoned small tree finch nests (2 out of 9 nests, numbers of *P. downsi* specimens ranged from 1 to 13, median 2). The only available blood source in such nests is the incubating female suggesting that they are also being parasitized by *P. downsi* larvae. Independent support for this comes from the finding that incubating female medium ground finches from parasitized nests showed higher *P. downsi*-specific antibody levels than females from parasite free nests^24^. Based on this knowledge about the negative effect of mosquitoes and *P. downsi* on Darwin’s finches we hypothesized that the behaviour of rubbing plants into feathers could have one or more possible effects: the plant extract might deter mosquitoes from biting; it might prevent adult *P. downsi* flies from entering the nest; it might protect incubating females from being bitten by *P. downsi* larvae or it might affect larval growth. In the light of these possible effects, we tested the repellent effect of *P. galapageium* on mosquitoes, on adult *P. downsi* flies and also its effect on the growth of *P. downsi* larvae.

## Results

### Repellent effect of *P. galapageium* on Mosquitoes

#### Field-experiment

We tested the repellent effect of *P. galapageium* on the local mosquito fauna in a field experiment using 17 human subjects, each of whom treated one leg and one arm with crushed *P. galapageium* leaves and left the other arm and leg untreated. During a 15-mintutes-exposure to mosquitoes in the field a total of 202 mosquito bites were counted. Significantly fewer mosquitoes than expected by random bit the limbs treated with *P. galapageium* (mean proportion of bites on treated limb ± standard error: 0.27 ± 0.11, Chi-squared Test for equal or given proportions, χ^2^ = 48.04, n = 17, p < 0.001)

#### Lab-experiment

**Figure 1:**
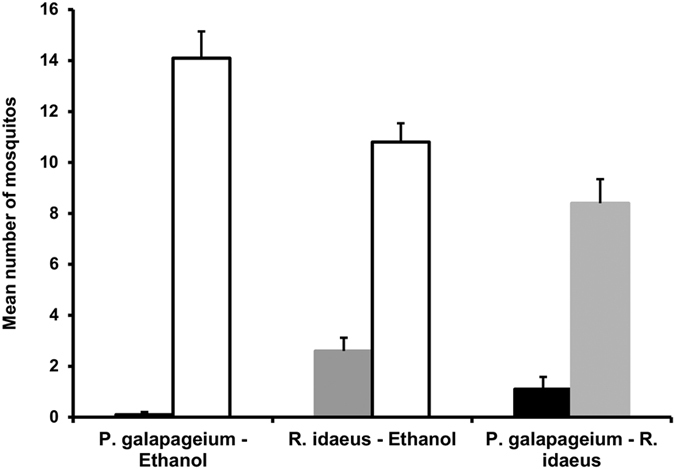
Repellent effect of *P. galapageium* on *Anopheles arabiensis*. Mean number (+SE) of mosquitoes which landed on the sausage treated with *P. galapageium* (black bar, only one mosquito landed in total) versus ethanol (white bar), *R. idaeus* (grey bar) versus ethanol (white bar) and which landed on the sausage treated with *P. galapageium* (black bar) versus *R. idaeus* extract (grey bar).

In the laboratory, we assessed the response of the mosquito *Anopheles arabiensis* to blood-filled sausages treated with an ethanol extract of *P. galapageium* leaves compared to two controls, one treated with ethanol and the second with an extract of *Rubus idaeus*. Significantly fewer mosquitoes than expected by random landed on the sausage treated with *P. galapageium* extract than on the ethanol-treated control sausage (mean proportion of mosquitos on ethanol-treated sausage ± standard error: 0.99 ± 0.03, Chi-squared Test for equal or given proportions, χ^2^ = 138.99, n = 10, p < 0.0001, Fig. 1, Table S2). The mosquitoes significantly avoided the *R. idaeus* extract when tested against the sausage treated with ethanol (mean proportion of mosquitos on ethanol-treated sausage ± standard error: 0.81 ± 0.12, Chi-squared Test for equal or given proportions, χ^2^ = 56.437, n = 10, p < 0.0001, Fig. 1, Table S2). However, significantly more mosquitoes landed on the sausage treated with *R. idaeus* when tested against the sausage treated with *P. galapageium* (mean proportion of mosquitos on *R. idaeus*-treated sausage ± standard error: 0.91 ± 0.09, Chi-squared Test for equal or given proportions, χ^2^ = 60.59, n = 10, p < 0.0001, Fig. 1, Table S2).

### Effect of *P. galapageium* on *Philornis downsi*

#### Effect on larval growth

**Figure 2:**
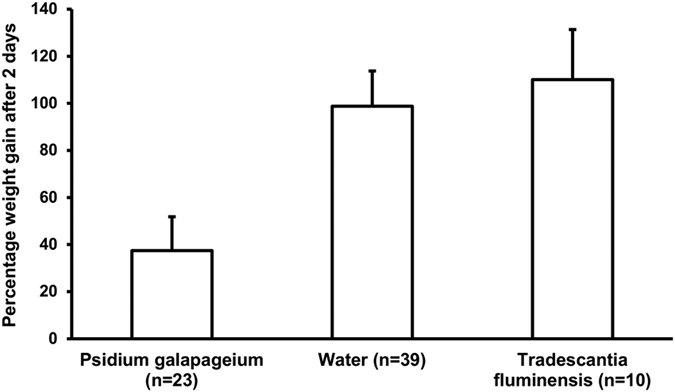
Effect of *P. galapageium* on larval growth of *P. downsi*. Mean (+SE) percentage of weight gain after two days of *P. downsi* larvae which fed from a blood source treated with mashed *P. galapageium* leaves, water or mashed *Tradescantia fluminensis* leaves, a non-repellent plant. Percentage weight gain was calculated as (“Weight after 2 days” − “Initial weight”)/“Initial weight” × 100.

We also measured the effect of *P. galapageium* leaves on larval growth (measured as weight after two days) compared to the two control treatments (water and *Tradescantia flumensis,* a plant which is not known to have a repellent effect) and included the initial weight of the larva as a co-variable in the model (linear model: F = 25.41, df = 68, p < 0.001 and adjusted R^2^ = 0.51). Larvae that fed on chicken blood through gauze treated with *P. galapageium* leaves had significantly lower weight gain compared to the weight gain of larvae in two control groups (Post-hoc comparison of group means adjusted for the covariate initial weight: Tukey’s honest significant difference (HSD) test, *P. galapageium* – Water, T_60_ = 2.83, p = 0.02; *P. galapageium* – *T. fluminensis*, T_31_ = 2.59, p = 0.02, Fig. 2).

#### Effect on adult flies

To test whether *P. galapageium* repels adults of *P. downsi* we used a Y-tube laboratory olfactometer. The flies could choose between entering the arm which delivered the odour of activated yeast, which is attractive to this species (see methods), and the other arm which delivered a combined odour of activated yeast and *P. galapageium* extract. Of 37 females tested, 18 chose the control arm (activated yeast only), five chose the *P. galapageium* treatment (activated yeast combined with *P. galapageium* extract) and 14 did not respond. The 23 flies that responded chose the control treatment significantly above chance (binomial test: p = 0.011).

### Coupled Gas Chromatographic-Electroantennographic Detection (GC-EAD)

**Figure 3:**
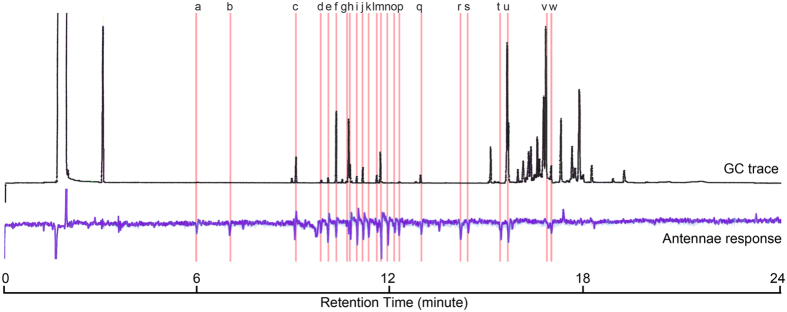
Representative GC-EAD analysis that tested the antennae of adult female *P. downsi* (lower trace) for responses to volatile components of *P. galapageium* leaf extracts (upper trace). Twenty-three compounds (indicated by vertical lines) consistently elicited responses from *P. downsi* antennae. Letters of the 23 antennally active compounds correspond to those in Table 2.

We conducted GC-EAD analysis to verify that the olfactory system of *P. downsi* includes receptors for volatile components of *P. galapageium* leaves. *Philornis downsi* antennae responded to 23 compounds in the *P. galapageium* extracts (Fig. 3), which are listed in Table 2 with their respective retention indices. The antennally active compounds are principally monoterpenoids and sesquiterpenes.

## Discussion

Our experiments suggest the repellent effect of *P. galapageium* on mosquitoes and adult *P. downsi* and show a growth inhibiting effect on *P. downsi* larvae. We recognize that the actual concentration of the plant volatiles applied by a bird to its feathers can be different than the concentration tested in our behavioural assays. In Y-tube bioassays, flies were behaviourally sensitive to one tenth equivalent amount of the leaf extract, suggesting that some of the tested leaf volatiles could maintain repellency at reasonably low concentrations. It is also possible that the human skin microbiome or the alcohol used in the blood sausage assays could have affected the perception of the mosquitoes. However, we were able to demonstrate that *P. galapageium* has a repellent effect using three different extraction methods (crushed leaves, alcohol extraction, dichloromethane extraction). The fact that consistent results were obtained with each method supports the conclusion that *P. galapageium* is repellent under a variety of background conditions. These findings are in line with our hypothesis that the behaviour of self-treating feathers with *P. galapageium* leaves confers a degree of protection from parasitism. These lab based experiments are good indirect evidence for this effect but further studies are needed to confirm that this behaviour does indeed provide this form of protection for Darwin’s finches.

Preening with *P. galapageium* may be considered an example of self-medication, which is defined as defence against parasites, pathogens, or both, by one species applying substances produced by another species^25^. Clayton and Wolf 25 suggest three aspects that characterise self-medication. Here we provide evidence for two aspects: (1) that the medical substance is deliberately contacted by the medicator and (2) that the substance has a negative effect on the parasite^25^. However, de Roode *et al*.^26^ point out that self-medication may not necessarily lead to a reduction of parasites. It could also enhance host fitness by increasing tolerance of infection. The third aspect suggested by both Clayton and Wolf 25 and de Roode *et al*.^26^ is, that the behaviour increases the fitness of the medicator. The repellent quality of *P. galapageium in situ* and evidence for increased fitness of the medicator as a result of using the substance remain to be tested.

Self-medication in the form of ingestion of medicinal plants is widespread in great apes (reviewed in ref. 27) but also in insects. For instance moths increase the ingestion of particular chemicals that are already in their diets^28^. Most examples of medicinal plant ingestion are forms of therapeutic self-medication in which diseased individuals change their behaviour in response to parasitic infection. In contrast, prophylactic self-medication is used by infected and uninfected individuals to prevent parasite infection often in response to high parasite risk^28^,^29^. For instance, several bird species (reviewed in ref. 30) and wood ants *Formica paralugubris*^29^ have been observed incorporating plant material with medicinal properties into their nests.

Topical application is a subcategory of self-medication which is rare (reviewed in ref. 25). While there are only few studies that report insecticidal effects of substances applied by birds which lead to a reduction of their ectoparasite load (reviewed in refs 12 and 25), we have shown that *P. galapageium* has a repellent rather than an insecticidal effect on adult *P. downsi* and on mosquitoes. Preening with this plant may therefore, prevent the parasites making contact with the birds in the first place rather than eliminating the insects after potentially detrimental contact has been made. Thus, it may represent a form of prophylactic self-medication. The effect on the larvae of *P. downsi* may be insecticidal or repellent. Ingestion of *P. galapageium* on the gauze could have an insecticidal effect and/or the larvae were repelled by the plant and did not ingest as much food as the control groups. Our current assay does not allow us to distinguish between these two possibilities.

Many plants produce secondary metabolites to protect against herbivores and pathogens. Several volatile components of *P. galapageium* that we have demonstrated that *P. downsi* can detect also occur in other plant species, which have been shown to have repellent effects on arthropods. For instance *Psidium guajava* produces volatiles that repel the sapsucking insect *Diaphorina citri*^31^,^32^,^33^. The volatile constituents of *P. guajava* that have repellent properties are the three part combination of limonene, α-pinene and β-pinene^33^. All three of these compounds are present in *P. galapageium* and elicited antennal responses in our GC-EAD analysis. Other components that induced antennal responses in our analysis include β-caryopyllene and linalool. β-Caryopyllene is one of the dominant components of *P. galapageium* and has been shown to have a repellent effect on mosquitoes of the genera *Armigeres*, *Culex* and *Aedes*^34^. Linalool has been found to act as a repellent against several species of mosquitoes of the genera *Aedes* and *Culex*^35^,^36^,^37^. It remains to be determined precisely which volatiles of *P. galapageium* repel *P. downsi* and local mosquito species, but as we have shown, *P. galapageium* contains multiple components that have been demonstrated to have repellent effects on a wide variety of arthropods. This suggests that the exploitation of this plant’s secondary metabolites by finches is effective.

In sensitive ecosystems such as those of the Galápagos Islands the use of insecticides is problematic due to effects on non-target organisms^38^ including natural enemies^39^, beneficial organisms^40^ and wildlife in general^41^,^42^. Thus, the discovery of the repellent effect of an endemic plant is potentially important for the conservation of threatened or endangered endemic bird populations suffering from non-native parasites and pathogens. Several studies have successfully reduced the number of *P. downsi* larvae in Darwin’s finch nests by using applied insecticides (e.g. refs 24,43 and 44). However, they all used substances that are potentially toxic to non-target organisms. Although numerous plant compounds are known for their repellent effects^45^,^46^ (and see above) the number of commercially available bio-insecticides is surprisingly low. Thus, the identification of the active compounds of *P. galapageium* might serve as basis for developing a novel plant-based repellent.

The behaviour reported in this paper is yet another example of exceptional behavioural flexibility and innovation in Darwin’s finches. Several definitions of innovation have been proposed (reviewed in ref. 47). According to Kummer and Goodall^48^ innovations can be an existing solution to a novel problem or a novel solution to an existing problem and can be based on already existing, completely new or modified behaviour patterns. In the current example, preening is an already existing behaviour and the novel aspect is the incorporation of an object into the behavioural sequence. It can be seen as a form of active anting, i.e. grabbing ants or other objects and rubbing them into the feathers. According to recent definitions, active anting is now accepted as a form of tool use^49^,^50^,^51^,^52^. Anting is taxonomically widespread in birds and thus might be an ancestral trait in Darwin’s finches but the use of *P. galapageium* for this purpose evolved after their ancestors colonised the islands.

We can only speculate how the use of *P. galapageium* leaves became incorporated into the sequence of preening. Increasing evidence that passerines have sufficient olfactory abilities to detect chemical cues (reviewed in ref. 53) raises the possibility that such cues could be used to select appropriate objects for anting^15^. Clark and Mason^54^,^55^ showed that starlings and brown-headed cowbirds could be conditioned to discriminate between different plant volatiles. The proximate mechanism by which preening with plants is initially reinforced might be that it either reduces disturbance by insects and/or reduces the itching sensation caused by insect bites. Whether *P. galapageium* has such properties still needs to be tested.

There is no direct evidence to indicate when the use of *P. galapageium* leaves emerged in Darwin’s finches. It is possible that this form of tool-use may have existed for a long time and has gone unnoticed despite the fact that several research groups have studied the behaviour of Darwin’s tree finches at the same study site for two decades. It is also possible that an existing behaviour has increased in frequency during recent years in response to the selection pressure exerted by novel mosquito-borne pathogens and the recently introduced parasite *P. downsi*.

## Methods

### Field Observations

Observations were made opportunistically during a field study on the influence of *Philornis downsi* on the breeding success of Darwin’s finches. This study was conducted at a study site (Los Gemelos, 0°37’34” S, 90°23’10” W) in the humid “Scalesia” forest on the Island of Santa Cruz, between January 11 - April 25 2012, January 9 - April 31 2014 and January 15 - May 30 2015. During this period *P. downsi* prevalence was very high. In 2012 all investigated nests with chicks of warbler finches (n = 46) and small tree finches (n = 37) were infested by *P. downsi* larvae. In 2014 and 2015 the prevalence was similar: 2014 warbler finch 97.9% (n = 96), small tree finch 95.8% (n = 72); 2015 warbler finch 94.1% (n = 51), small tree finch 90.2% (n = 51). However, we have no data on the prevalence of mosquitoes in the study area.

### Repellent effect of *P. galapageium* on Mosquitoes

#### Field-experiment

In the field, the 17 human subjects (10 men, 7 women between 21 and 56 years old, see Table S1) treated one leg and one arm with ten crushed *P. galapageium* leaves each and left the other arm and leg untreated. The side of treatment was assigned randomly. Participants were standing while the experiment. The number of mosquitoes which were observed biting within 15 min of exposure on treated and non-treated limbs was counted. The experiment was carried out on the 9^th^ of March 2016 at 6:33 pm (participants 1–9) and on the 18^th^ of March 2016 at 6:30 pm (participants 10–17) at the Charles Darwin Station, Puerto Ayora, Galápagos. All participants were given the instruction not to shower two hours previous to testing and not to use repellents. To test whether the choice of the mosquitoes was different from random we used a “test of equal or given proportions” prob.test. library stats, R 3.2.2.,)^56^. Sample size was defined by the number of treated subjects (17), which were tested only once to avoid pseudoreplication.

#### Lab-experiment

The experiment was conducted at the Insect Pest Control Laboratory (IPCL) of the Joint FAO/IAEA Division, Seibersdorf, Austria. The *Anopheles arabiensis* used in this study originated from Dongola in the northern state of Sudan in 2005 and have been maintained and reared since then in the laboratory (for details see ref. 56). Adults were kept in a climate-controlled room maintained at a temperature of 27 ± 1 °C and 60 ± 10% relative humidity on a 12:12 h photoperiod, including dusk (1 h) and dawn (1 h). For the experiments, 20 five-day old female *An. arabiensis* were placed in a 30 cm cubic insect cage (Megaview Science Education Services Co., Ltd., Taiwan) the day before the experiment started and fed with 5% sugar-solution.

To test the repellent effect of *P. galapageium*, collagen sausage casings (Edicas 23NC, FIBRAN S.A., Girona, Spain) filled with 50 ml of defibrinated bovine blood heated at 38–39 °C were used. Each sausage was moistened with extract of *P. galapageium* or control plant or with their respective solvent. To prepare the *P. galapageium* extract, dried and ground *P. galapageium* leaves, collected at Los Gemelos, Santa Cruz, Galápagos, were put in 70% ethanol for one week at a weight ratio of 1:10 ground leaves to ethanol. After seven days the extract was filtered and preserved in a sealed glass bottle at room temperature until use.

During the experiment, two sausages were simultaneously presented to the female mosquitoes. One sausage was moistened with repellent while the second sausage was moistened with the control plant or solvent. The sausages were placed in the cage in parallel separated by 10 cm and the experimenter blew 5 times into the cage to motivate the mosquitoes to move. The following combination was tested: *P. galapageium* extract against ethanol. To test whether the application of a plant extract has a repellent effect *per se*, we introduced an additional control using *Rubus idaeus* as a plant species that is not known to have repellent properties and is closely related to the invasive *Rubus niveus* which was very abundant in our study area. We prepared the *R. ideaeus* extract in the same way as the *P. galapageium* extract and tested it against the solvent ethanol. Contrary to our expectation *R. ideaus* had a repellent effect compared to ethanol. In a subsequent experiment we thus tested *P. galapageium* extract against *R. idaeus* extract to assess which of the two plants had the stronger repellent effect. To measure the repellent effect we counted the number of mosquitoes sitting on each sausage after 60 seconds of exposure and repeated this every minute, for nine more minutes. The results presented are based on the number of mosquitoes, which landed on the treated sausage (*P. galapageium, R.ideaus*) after the first 60 seconds of exposure to the sausages. However, in the subsequent 9 min the percentage of mosquitoes that landed on the sausage treated with *P. galapageium* was similar to the results from the first 60 seconds (first 60 sec: median 3.3%, range 0–41.5%; subsequent 9 min.: median 0%, range 0–45%).

We conducted ten replicates for each condition. For each replicate new sausages and new cages with different mosquitoes were used. The side of treatment and control was assigned randomly. The number of female mosquitoes, which were still alive when the experiment started, ranged from 15 to 20 (median 19) in each trial. During the first 60 seconds the percentage of mosquitoes which landed on either of the sausages in all trials and conditions ranged from 29–100% (median 66%). All data are shown in Table S2. Again we used a test of equal or given proportion to estimate whether the choice of the mosquitoes in each comparison *(P.galapageium* – ethanol, *R. idaeus* – ethanol, *P. galapageium* – *R. idaeus)* was different from random (prob.test. library stats, R 3.2.2.^57^). We have adjusted the significance level to p = 0.017 using a Bonferroni correction

### Repellent effect of *P. galapageium* on *P. downsi*

#### Effect on larval growth

In a laboratory experiment, we tested whether *P. galapageium* leaves reduce larval growth of *P. downsi*. Larvae were collected from the study site “Los Gemelos” between 07^th^ February and 1^st^ April 2014, which is the main breeding season of Darwin’s finches (Table S3). We only used second instar, small to medium sized larvae from two different size classes: <0.5 cm small larvae, 0.5 cm–1.0 cm medium larvae. Larvae were assigned randomly to test and control group and raised in the laboratory at the Charles Darwin Station, Santa Cruz, Galápagos, and fed with chicken blood that was applied to a piece of cotton, which was tamped into a short piece of plastic drinking straw. The tip of the straw was covered with gauze. One group had to feed through gauze on which one drop of crushed *P. galapageium* leaves was applied. To produce the crushed leaves, four leaves and 1 ml of water were put into an Eppendorf tube (2 ml) and crushed with metal forceps. The other group had to feed through gauze that was treated with water only. Blood and treatment was renewed daily. To test whether the application of a plant extract had a repellent effect *per se*, we introduced an additional control group with crushed *Tradescantia fluminensis* leaves, a plant species present on the Galápagos Islands that is not known to have repellent properties. Biosafety regulations severely limit the movement of plants into and out of the Galápagos, therefore we used a different control plant in this lab experiment which was conducted in the Galápagos than that used in the lab-experiment on the repellent effect of *P. galapageium* on mosquitoes described above which was conducted in Austria. We weighed the larvae before the experiment and after 2 days of treatment and calculated the percentage of weight gain as “weight after 2 days” − “initial weight”)/“initial weight” × 100. Raw data are presented in Table S3. To analyse the effect of *P. galapageium* leaves on larval growth compared to the two control treatments (water and *T. fluminensis*) we calculated a linear model with weight after 2 days as predicted variable and treatment and initial weight as predictor variable. We calculated the model with interactions, but since none was significant we excluded them from the final model. We omitted one larva of the *P. galapageium* treatment group as its weight decreased by the power of ten, likely representing a typing error in data entering. However, the results did not change without this outlier. Weights were log-transformed. The post-hoc tests were done with the R-package multcomp^58^.

#### Effect on adult flies

We used a Y-tube laboratory olfactometer to assess the repellent effect of *P. galapageium* extract on the attractiveness of activated yeast to *P. downsi*. Olfactometer arms were 30 cm × 6 cm dia., and the angle between the arms was 70°. The y-tube was positioned vertically and a fluorescent lamp with dual bulbs (1.2 m) was centred 50 cm above the olfactometer parallel to a line connecting the ends of the olfactometer arms. Charcoal filtered air was introduced (1.0 L/min) into Erlenmeyer flasks (125 mL) fitted with two-hole rubber stoppers for incurrent and excurrent air flows. Effluent from the flasks was transferred to the olfactometer arms through plastic tubing (5 mm i.d.) and connected to a glass adapter with a barbed fitting on one end and a ground glass fitting (6 cm dia.) on the other end. The positions of the control and treatment were switched after every five trials. Adult *P. downsi* were released in the centre arm of the olfactometer and allowed to fly upwards towards the light and odour sources. A positive or negative response was recorded if the fly stayed in the distal half of the treatment or control arm for at least one minute. The majority of responding flies chose one arm within ten seconds.

The first experiment was intended to establish the attractiveness of yeast and the veracity of the olfactometer assay. The test stimulus was activated yeast (0.5 g baker’s yeast, 2.5 g sugar, 30 mL water) and the control was deionized water (30 mL). Of the 38 assays of *P. downsi* males 13 chose the yeast treatment, 9 chose the water control and 16 were unresponsive. The 22 responsive males showed no significant preference for the yeast treatment (binomial test: p = 0.12). Of the 60 assays of *P. downsi* females 25 chose the yeast treatment, 11 chose the water control and 24 were unresponsive. The 36 responsive females showed a significant preference for the yeast treatment (binomial test: p = 0.01). Thus, only female flies were used for the following experiment.

The purpose of the second experiment was to determine the repellent effect of *P. galapageium* extracts. Leaves of *P. galapageium* were collected in the Los Gemelos highlands of Santa Cruz Island on 26 February, 2015, transported to the Charles Darwin Research Station, Puerto Ayora, and stored at 4 °C. Leaf extracts were made within 24 hours by placing four crushed *P. galapageium* leaves in a vial (4 mL) with dichloromethane (4 mL) for 10 minutes and then decanting the solvent extract into a fresh vial. Thus, each assay used 0.1 *P. galapageium* leaf equivalents (4 leaves in 4 mL DCM = a concentration of 1.0 leaf/mL; the 100 uL aliquots used in the assays would represent 0.1 leaf eq.). It is impossible to say how much volatile material a finch applies to its feathers, but based on our field observations, a tenth of one leaf is reasonably close to the amount used by finches. In Syracuse, extracts were stored at −60 °C until use. An aliquot (100 μL) of the extract was placed on a filter paper strip inside a glass tube that was inserted in the plastic tubing between the Erlenmeyer flask and the olfactometer arm.

Controls consisted of an equal volume of methylene chloride on a filter paper strip. The Erlenmeyer flasks of both the treatment and control contained activated yeast as described above.

### Coupled Gas Chromatographic-Electroantennographic Detection (GC-EAD)

In a further experiment coupled Gas Chromatographic-Electroantennographic Detection was carried out to give insight into which chemical compounds of *P. galapageium* leaves can be detected by adult *P. downsi*. Coupled GC-EAD analysis was performed using a gas chromatograph (Hewlett-Packard 5890 Series II) equipped with a capillary column (HP5-MS, 30 m × 0.25 mm ID, 0.25 μm film thickness; Agilent Technologies, Wilmington, DE, USA) in splitless mode with 1 min sampling. The oven temperature was programmed for 5 min at 40 °C, then increased at 15 °C/min to 250 °C, and then held for 5 min. The injector temperature was 250 °C. Helium was the carrier gas at a flow rate of 1 mL/min. The column effluent was split 1:1 in the oven via a glass Y-connector with nitrogen make-up gas (8 mL/min) introduced through a second glass Y-connector. One arm of the splitter led to the flame ionization detector (FID) (260 °C) and the other to a heated transfer line (260 °C) (Agilent Technologies, Wilmington, DE, USA). The EAD effluent was introduced into a cooled (5 °C) humidified air stream (1 L/min) directed toward the antennae of the mounted fly.

Whole head preparations were made of individual flies, age 3–6 d, for GC-EAD analysis, as described previously from similar studies with other flies^59^,^60^,^61^. The head was excised and the antennae were positioned between two gold-saline (Drosophilaringer solution; 46 mmol NaCl, 182 mmol KCl, 3 mmol CaCl2, and 10 mmol Tris HCl at pH 7.2) electrode micropipettes in an acrylic holder. The output signal from the antenna was amplified (10×) by a custom high input impedance DC amplifier and recorded on an integrator-recorder.

For the GC-EAD analysis of *P. galapageium* leaf extracts, a total of four antennal pairs from two female and two male flies (1–2 replicated runs/pair) were tested.

To identify the chemical compounds present in the *P. galapageium* leaf extract to which *P. downsi* antennae were responding, leaf extracts were analysed by coupled gas chromatography-mass spectrometry (GC-MS; Agilent 7890A GC interfaced to a 5975 mass selective detector in EI mode, 70 eV; Agilent Technologies, Santa Clara, CA, USA). The GC column, temperature program and carrier gas were the same as those used for GC-EAD. Compound identifications were based on matches with spectra in the NIST/EPA/NIH Mass Spectral Library Version 2.0f (2009) and retention index (Table 2).

## Ethical Statement

The study was conducted in the protected areas of the Galápagos National Park. Permission to conduct this study was granted by the Galápagos National Park and the Charles Darwin research station (Project: PC-54-11 and Project: PC-02-14; Permit Nr. PR.CDS.ACI.P01.R02). For the experiment involving human subjects all experimental protocols were approved by the Charles Darwin research station (FCD-DEJ-16-167), and informed consent was obtained from all subjects. All methods were carried out in accordance with the approved guidelines.

## Additional Information

**How to cite this article**: Cimadom, A. *et al*. Darwin’s finches treat their feathers with a natural repellent. *Sci. Rep.* **6**, 34559; doi: 10.1038/srep34559 (2016).

## Supplementary Material

Supplementary Video S1

Supplementary Information

## Figures and Tables

**Table 1:** Observations of topical application of *P. galapageium* leaves by different Darwin’s finch species.

Species	Year	Method^1^
Warbler finch (*Certhidea olivacea*)	2012	Sponge
Warbler finch (*Certhidea olivacea*)	2012	Lotion
Warbler finch (*Certhidea olivacea*)	2012	Sponge
Small tree finch (*Camarhynchus parvulus*)	2012	Lotion
Small tree finch (*Camarhynchus parvulus*)	2012	Lotion
Medium ground finch (*Geospizia fortis*)	2014	Sponge and lotion
Small ground finch (*Geospizia fuliginosa*)	2014	Sponge and lotion
Small ground finch (*Geospizia fuliginosa*)	2014	Sponge and lotion
Warbler finch (*Certhidea olivacea*)	2015	Lotion
Small tree finch (*Camarhynchus parvulus*)	2015	Sponge

^1^Sponge method: bird threads piece of leaf through the feathers, lotion method: bird chews leave first and applies resulting mixture of saliva and mashed leaf.

**Table 2:** *P. galapageium* leaf compounds eliciting GC-EAD responses from *P. downsi.*

	Chemicals	Retention index
a	3-Hexenal	
b	Diacetone alcohol	845
c	α-Pinene	936
d	β-Pinene	980
e	β-Myrcene	993
f	α-Phellandrene	1007
g	D-Limonene	1034
h	Eucalyptol	1036
i	β-Ocimene	1051
j	γ-Terpinene	1064
k	Guaiacol	1072
l	α-Terpinolen	1093
m	Linalool	1101
n	unknown	1112
o	unknown	1128
p	unknown	1146
q	α-Terpineol	1197
r	unknown	1296
s	unknown	1318
t	Eremophilene	1413
u	β-Caryophyllene	1442
v	(*E*)-Nerolidol	1572
w	unknown	1584
