# Supplementary material for: Darwin’s finches treat their feathers with a natural repellent
Source: Sci Rep. 2016 Oct 10;6:34559. doi: 10.1038/srep34559 (PMC5056383; doi:10.1038/srep34559)
Supplement: Supplementary Information [file srep34559-s2.pdf]

Supplementary Material for

# Darwin's finches treat their feathers with a natural repellent

Arno Cimadom[1+], Charlotte Causton[2], Dong H. Cha[3], David Damiens[4], Birgit Fessl[2], Rebecca Hood-Nowotny[5], Piedad Lincango[2], Alejandro E. Mieles[3], Erwin Nemeth[6], Elizabeth M. Semler[3], Stephen A. Teale[3], Sabine Tebbich[1+*]

**Affiliations:**

[1]Department of Behavioural Biology, University of Vienna, Vienna, Austria

[2]Charles Darwin Foundation, Puerto Ayora, Santa Cruz Island, Galápagos, Ecuador

[3]Department of Environmental and Forest Biology, College of Environmental Science and Forestry, State University of New York, Syracuse, USA

[4]Insect Pest Control Laboratory, Joint FAO/IAEA Division of Nuclear Techniques in Food and Agriculture, International Atomic Energy Agency, Vienna, Austria

[5]Health and Environment Department, AIT Austrian Institute of Technology GmbH, Tulln, Austria

[6]BirdLife Austria, Vienna, Austria

**Supplementary Video S1**

Warbler finch (*Certhidea olivacea*) tearing the tips of leaves of the endemic Guayabillo tree, *Psidium galapageium*, chewing them and rubbing them on its feathers. The video was recorded on the 24.02.2016, 6:30 am, at Los Gemelos, Santa Cruz Island, Galapagos.

**Supplementary Table S1**

Results of the repellence test of *Psidium galapageium* against mosquitoes in the field. Participants 1 – 9 were tested on March 9th 2016, 6.33pm, participants 11 – 17 on March 18th 2016, 6.30 pm. All tests were conducted at the Charles Darwin Station, Puerto Ayora, Galapagos, Ecuador.

| | | | | Number of bites on | |
|---|---|---|---|---|---|
| **Participant** | **Gender** | **Age** | **Treated side** | ***P. galapageium* treated limbs** | **untreated limbs** |
| 1 | female | 22 | left | 1 | 8 |
| 2 | male | 27 | left | 2 | 5 |
| 3 | female | 24 | right | 2 | 6 |
| 4 | male | 27 | left | 4 | 3 |
| 5 | male | 27 | right | 3 | 4 |
| 6 | male | 28 | left | 0 | 2 |
| 7 | male | 30 | right | 2 | 8 |
| 8 | female | 22 | right | 1 | 2 |
| 9 | female | 32 | right | 5 | 5 |
| 10 | male | 31 | right | 6 | 9 |
| 11 | male | 23 | left | 4 | 17 |
| 12 | female | 21 | right | 1 | 6 |
| 13 | male | 56 | right | 3 | 9 |
| 14 | male | 28 | right | 1 | 4 |
| 15 | male | 40 | left | 9 | 23 |
| 16 | female | 21 | left | 16 | 22 |
| 17 | female | 30 | left | 0 | 9 |

**Supplementary Table S2**

Data of the lab-experiment assessing the response of the mosquito *Anopheles arabiensis* to blood-filled sausages treated with an ethanol extract of *P. galapageium* leaves compared to two controls (ethanol and extract of *Rubus idaeus*). Table presents the number of tested mosquitoes (in cage) and the number of mosquitos which were sitting on the treatment- and control-sausage after 60 seconds of exposure for each trial and test. The percentage of mosquitos on treatment-sausage is based on the total number of mosquitos which landed on either of the sausage.

| | | Number of mosquitos | | | % Mosquitos |
|---|---|---|---|---|---|
| **Test** | **Trial** | **in cage** | **on** | **on** | **on** |

| | | | treatment-sausage | control-sausage | treatment-sausage |
|---|---|---|---|---|---|
| *P. galapageium* - Ethanol | 1 | 17 | 0 | 12 | 0 |
| *P. galapageium* - Ethanol | 2 | 19 | 0 | 12 | 0 |
| *P. galapageium* - Ethanol | 3 | 17 | 0 | 14 | 0 |
| *P. galapageium* - Ethanol | 4 | 15 | 0 | 8 | 0 |
| *P. galapageium* - Ethanol | 5 | 20 | 0 | 20 | 0 |
| *P. galapageium* - Ethanol | 6 | 17 | 0 | 15 | 0 |
| *P. galapageium* - Ethanol | 7 | 19 | 1 | 13 | 8 |
| *P. galapageium* - Ethanol | 8 | 18 | 0 | 15 | 0 |
| *P. galapageium* - Ethanol | 9 | 20 | 0 | 18 | 0 |
| *P. galapageium* - Ethanol | 10 | 19 | 0 | 14 | 0 |
| *R. diaeus* - Ethanol | 1 | 19 | 3 | 13 | 23 |
| *R. diaeus* - Ethanol | 2 | 15 | 5 | 10 | 50 |
| *R. diaeus* - Ethanol | 3 | 20 | 5 | 8 | 63 |
| *R. diaeus* - Ethanol | 4 | 20 | 4 | 9 | 44 |
| *R. diaeus* - Ethanol | 5 | 20 | 2 | 14 | 14 |
| *R. diaeus* - Ethanol | 6 | 20 | 2 | 10 | 20 |
| *R. diaeus* - Ethanol | 7 | 20 | 2 | 12 | 17 |
| *R. diaeus* - Ethanol | 8 | 21 | 2 | 12 | 17 |
| *R. diaeus* - Ethanol | 9 | 20 | 0 | 7 | 0 |
| *R. diaeus* - Ethanol | 10 | 18 | 1 | 13 | 8 |
| *P. galapageium* - *R. diaeus* | 1 | 15 | 0 | 7 | 0 |
| *P. galapageium* - *R. diaeus* | 2 | 21 | 0 | 8 | 0 |
| *P. galapageium* - *R. diaeus* | 3 | 20 | 1 | 7 | 14 |
| *P. galapageium* - *R. diaeus* | 4 | 18 | 0 | 7 | 0 |
| *P. galapageium* - *R. diaeus* | 5 | 17 | 0 | 5 | 0 |
| *P. galapageium* - *R. diaeus* | 6 | 20 | 1 | 12 | 8 |
| *P. galapageium* - *R. diaeus* | 7 | 19 | 5 | 11 | 45 |
| *P. galapageium* - *R. diaeus* | 8 | 20 | 1 | 14 | 7 |
| *P. galapageium* - *R. diaeus* | 9 | 20 | 2 | 8 | 25 |
| *P. galapageium* - *R. diaeus* | 10 | 20 | 1 | 5 | 20 |

**Supplementary Table S3**

Initial weight and weight after 2 days of *P. downsi* larvae in the three different rearing treatments (water, *Psidium galapageium* and *Tradescantia fluminensis*). Percentage weight gain was calculated as ("Weight after 2 days" – "Initial weight") / "Initial weight" x 100.

| Treatment | Date | Initial weight [g] | Weight after 2 days [g] | % weigth gain |
|---|---|---|---|---|
| Water | 07.02.2014 | 0.0185 | 0.0338 | 82.7 |
| Water | 07.02.2014 | 0.0197 | 0.0443 | 124.9 |
| Water | 07.02.2014 | 0.0170 | 0.0234 | 37.6 |

| | | | | |
|---|---|---|---|---|
| Water | 07.02.2014 | 0.0154 | 0.0265 | 72.1 |
| Water | 09.02.2014 | 0.0059 | 0.0174 | 194.9 |
| Water | 14.02.2014 | 0.0167 | 0.0131 | -21.6 |
| Water | 14.02.2014 | 0.0118 | 0.0079 | -33.1 |
| Water | 14.02.2014 | 0.0173 | 0.0177 | 2.3 |
| Water | 14.02.2014 | 0.0138 | 0.0078 | -43.5 |
| Water | 14.02.2014 | 0.0097 | 0.0059 | -39.2 |
| Water | 14.02.2014 | 0.0126 | 0.0225 | 78.6 |
| Water | 14.02.2014 | 0.0164 | 0.0129 | -21.3 |
| Water | 15.02.2014 | 0.0180 | 0.0150 | -16.7 |
| Water | 15.02.2014 | 0.0091 | 0.0136 | 49.5 |
| Water | 15.02.2014 | 0.0088 | 0.0152 | 72.7 |
| Water | 15.02.2014 | 0.0146 | 0.0163 | 11.6 |
| Water | 21.02.2014 | 0.0278 | 0.0692 | 148.9 |
| Water | 21.02.2014 | 0.0354 | 0.1157 | 226.8 |
| Water | 21.02.2014 | 0.0404 | 0.1025 | 153.7 |
| Water | 25.02.2014 | 0.0238 | 0.0456 | 91.6 |
| Water | 25.02.2014 | 0.0255 | 0.0493 | 93.3 |
| Water | 25.02.2014 | 0.0244 | 0.0531 | 117.6 |
| Water | 27.02.2014 | 0.0375 | 0.0395 | 5.3 |
| Water | 27.02.2014 | 0.0161 | 0.0416 | 158.4 |
| Water | 27.02.2014 | 0.0084 | 0.0289 | 244.0 |
| Water | 27.02.2014 | 0.0413 | 0.0643 | 55.7 |
| Water | 27.02.2014 | 0.0081 | 0.0288 | 255.6 |
| Water | 06.03.2014 | 0.0406 | 0.0728 | 79.3 |
| Water | 06.03.2014 | 0.0296 | 0.0593 | 100.3 |
| Water | 06.03.2014 | 0.0072 | 0.0144 | 100.0 |
| Water | 28.03.2014 | 0.0130 | 0.0365 | 180.8 |
| Water | 28.03.2014 | 0.0128 | 0.0345 | 169.5 |
| Water | 28.03.2014 | 0.0142 | 0.0539 | 279.6 |
| Water | 28.03.2014 | 0.0073 | 0.0337 | 361.6 |
| Water | 01.04.2014 | 0.0087 | 0.0175 | 101.5 |
| Water | 01.04.2014 | 0.0162 | 0.0252 | 55.6 |
| Water | 01.04.2014 | 0.0108 | 0.0233 | 116.0 |
| Water | 01.04.2014 | 0.0123 | 0.0275 | 123.8 |
| Water | 01.04.2014 | 0.0119 | 0.0215 | 81.0 |
| *Psidium galapageium* | 07.02.2014 | 0.0237 | 0.0180 | -23.8 |
| *Psidium galapageium* | 09.02.2014 | 0.0107 | 0.0127 | 18.7 |
| *Psidium galapageium* | 09.02.2014 | 0.0163 | 0.0107 | -34.4 |
| *Psidium galapageium* | 09.02.2014 | 0.0288 | 0.0389 | 35.1 |
| *Psidium galapageium* | 14.02.2014 | 0.0239 | 0.0188 | -21.3 |
| *Psidium galapageium* | 14.02.2014 | 0.0083 | 0.0039 | -53.0 |
| *Psidium galapageium* | 14.02.2014 | 0.0115 | 0.0085 | -26.1 |
| *Psidium galapageium* | 14.02.2014 | 0.0077 | 0.0052 | -32.5 |

| | | | | |
|---|---|---|---|---|
| *Psidium galapageium* | 15.02.2014 | 0.0166 | 0.0226 | 36.1 |
| *Psidium galapageium* | 15.02.2014 | 0.0311 | 0.0262 | -15.8 |
| *Psidium galapageium* | 15.02.2014 | 0.0225 | 0.0182 | -19.1 |
| *Psidium galapageium* | 15.02.2014 | 0.0278 | 0.0367 | 32.0 |
| *Psidium galapageium* | 15.02.2014 | 0.0094 | 0.0122 | 29.8 |
| *Psidium galapageium* | 15.02.2014 | 0.0183 | 0.0244 | 33.3 |
| *Psidium galapageium* | 15.02.2014 | 0.0156 | 0.0140 | -10.3 |
| *Psidium galapageium* | 21.02.2014 | 0.0154 | 0.0448 | 190.9 |
| *Psidium galapageium* | 21.02.2014 | 0.0265 | 0.0752 | 183.8 |
| *Psidium galapageium* | 25.02.2014 | 0.0196 | 0.0453 | 131.1 |
| *Psidium galapageium* | 25.02.2014 | 0.0374 | 0.0605 | 61.8 |
| *Psidium galapageium* | 25.02.2014 | 0.0194 | 0.0427 | 120.1 |
| *Psidium galapageium* | 27.02.2014 | 0.0218 | 0.0419 | 92.2 |
| *Psidium galapageium* | 27.02.2014 | 0.0633 | 0.0952 | 50.4 |
| *Psidium galapageium* | 27.02.2014 | 0.0281 | 0.0512 | 82.2 |
| *Tradescantia fluminensis* | 28.03.2014 | 0.0120 | 0.0221 | 84.4 |
| *Tradescantia fluminensis* | 28.03.2014 | 0.0110 | 0.0180 | 64.2 |
| *Tradescantia fluminensis* | 28.03.2014 | 0.0064 | 0.0196 | 206.3 |
| *Tradescantia fluminensis* | 28.03.2014 | 0.0118 | 0.0211 | 79.1 |
| *Tradescantia fluminensis* | 28.03.2014 | 0.0088 | 0.0088 | 0.0 |
| *Tradescantia fluminensis* | 01.04.2014 | 0.0083 | 0.0268 | 222.9 |
| *Tradescantia fluminensis* | 01.04.2014 | 0.0132 | 0.0301 | 128.3 |
| *Tradescantia fluminensis* | 01.04.2014 | 0.0142 | 0.0328 | 131.5 |
| *Tradescantia fluminensis* | 01.04.2014 | 0.0163 | 0.0268 | 64.4 |
| *Tradescantia fluminensis* | 01.04.2014 | 0.0129 | 0.0283 | 119.6 |
